# Two protocols using fluralaner for *Rhipicephalus microplus* strategic control on taurine cattle in a tropical region

**DOI:** 10.1186/s13071-023-06107-2

**Published:** 2024-01-08

**Authors:** Lidia Mendes de Aquino, Dina Maria Beltran Zapa, Daniel de Castro Rodrigues, Tom Strydom, Siddhartha Torres, Lorena Lopes Ferreira, Francisco Barufi, Heitor Oliveira Arriero de Amaral, Fernando de Almeida de Borges, Tiago Gallina, Rafael Paranhos de Mendonça, Vando Edésio Soares, Caio Marcio Oliveira Monteiro, Welber Daniel Zanetti Lopes

**Affiliations:** 1 Center of Veterinary Parasitology, School of Veterinary Science and Animal Science, Federal University of Goiás, Goiânia, Goiás Brazil; 2 MSD Animal Health, São Paulo, Brazil; 3 MSD Animal Health, 20 Spartan Road, Isando, Kempton Park, 1619 South Africa; 4 2 Giralda Farms, Merck Animal Health, Madison, NJ 07940 USA; 5 Department of Preventive Veterinary Medicine, School of Veterinary Medicine, Federal University of Minas Gerais, Belo Horizonte, Minas Gerais Brazil; 6 Department of Veterinary Medicine, Federal University of Mato Grosso Do Sul, Campo Grande, Mato Grosso Do Sul Brazil; 7 Federal University of Pampa, Uruguaiana, Brazil; 8 University of Franca, Franca, São Paulo Brazil; 9 University of Brazil, Descalvado, São Paulo Brazil; 10 Department of Biosciences and Technology, Institute of Tropical Pathology and Public Health, Federal University of Goiás, Goiânia, Goiás Brazil

**Keywords:** Cattle tick, Strategic control, Isoxazolines

## Abstract

**Background:**

The present study aimed to evaluate the effects of different treatment strategies using a new commercial formulation containing pour-on fluralaner on *Rhipicephalus microplus *infestation in cattle and in pastures in a tropical climate region where up to five generations of this tick species can occur per year.

**Methods:**

Forty-five naturally infested cattle were divided into three experimental groups: T01, treated with fluralaner (2.5 mg/kg) pour-on every 42 days; T02, the cattle received the first treatment with fluralaner on Day 0 but the next treatment involved a weekly visual evaluation; T03, control, received palliative treatment with a spray formulation when the group mean was ≥ 30 ticks. Counts of female *R. microplus* were performed weekly until day 343, and larval counts on pasture were performed on Days 0, 30, and 60 and every 30 days until Day 330.

**Results:**

Using fluralaner, six applications were performed in Group T01, and four were performed in Group T02. In the control group (T03), it was necessary to perform eight palliative acaricide treatments with the spray formulation. The animals in T01 and T02 showed lower mean tick counts (*p* ≤ 0.05) than the control group (T03) on 28 and 27 of the 49 evaluated dates, respectively. In the paddock where the animals were kept as controls, the *R. microplus* larvae counts increased to 1458. In the paddocks where the animals were treated with fluralaner, the number was ≤ 19 per paddock during the study.

**Conclusions:**

The different strategic treatment protocols performed with pour-on fluralaner (2.5 mg/kg) over a year in taurine cattle in a tropical region with a history of up to five annual generations of cattle ticks were effective, maintaining levels of *R. microplus* infestations in animals and in pastures close to zero in most of the study. Depending on the retreatment criterion adopted, the number of applications per year may be lower, resulting in a reduction in the mean cost of acaricide treatment per year and lower exposure of *R. microplus* populations to the active ingredient, resulting in lower resistance and selection pressure.

**Graphical Abstract:**

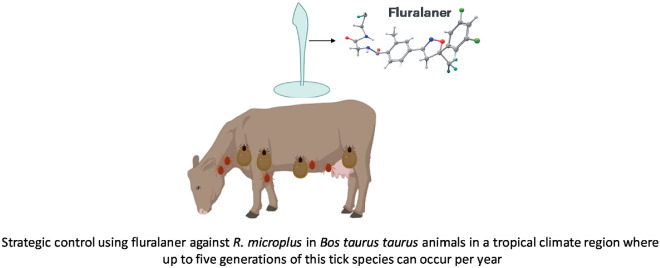

## Background

The cattle tick, *Rhipicephalus microplus*, is present in tropical and subtropical regions of the world, triggering significant losses of cattle worldwide. In Brazil alone, estimates point to losses of approximately US$ 3.24 billion per year [1, 2]. Although studies have evaluated alternative methods of control with fungal [3] and entomopathogenic nematodes [4], vaccines [5] and management measures [6], the main tool adopted against this ectoparasite is still the use of synthetic chemicals [7]. Despite the resistance of tick populations to the commercially available acaricides, a new chemical class used to control *R. microplus* was not launched for approximately 30 years [8]. The isoxazoline family was introduced to the market in 2014 and was the greatest innovation in the antiparasitic market of the twenty-first century. The active ingredient in this chemical class is fluralaner, which until then was available only for the control of ectoparasites in companion animals and birds [9–12].

The strategic control of cattle ticks is performed through acaricide applications at specific times and aims to maintain acceptable levels of infestation in animals and pastures, without the cattle showing clinical or productive losses due to parasitism. To devise a strategic treatment protocol, it is essential to know the number of annual generations that *R. microplus* can complete in a particular region, in addition to understanding the population dynamics of the ectoparasite. Based on the number of annual generations of the tick, it is possible to optimize the number of treatments required throughout the year and, with the population dynamics data, understand which generations may result in more or less intense levels of cattle and pasture infestation. With global warming, climate changes have occurred, such as the increase in annual mean temperatures in certain regions. In turn, this has resulted in an increase in the number of population peaks and annual generations that this ectoparasite can complete, reaching up to five generations in some geographic region [13–16]. In these regions, the challenge of tick control is greater, with the need to perform more treatments per year than in regions where there are only three generations per year [17]. In addition, studies have shown that the interval between acaricide applications tends to decrease during the period of the year when the level of parasitism by *R. microplus* is higher [18].

Another factor that must be considered when defining the best strategy to control *R. microplus* is the level of susceptibility of cattle to this ectoparasite. European breeds are more susceptible to parasitism than breeds of Indian origin, which is mainly explained by the immunological profile of each breed and their resistance to infestation, resulting from the coevolution of the tick with the breeds of Indian origin on the Asian continent [19–22]. In tropical regions with more annual generations of *R. microplus*, control of this tick species becomes more challenging on farms with taurine breeds [23]. Thus, the present study aimed to evaluate the effects of different treatment strategies and retreatment with a new commercial formulation containing pour-on fluralaner on the infestation of *R. microplus* in cattle and in pastures in a tropical climate region where up to five generations of this tick species can occur per year.

## Methods

### Location of the study

This experiment was conducted from November 2020 to October 2021 on a farm in São Paulo, located in the municipality of São José do Rio Pardo, state of São Paulo, Brazil (latitude 21°35ʹ44″ south; longitude 46°53ʹ19″ west; mean elevation: 676 m). This locality is in a region with a tropical climate dominated by the Cerrado biome, with two well-defined seasons: rainy summers (October to April), with mean annual rainfall of 1410 to 1430 mm, and dry winters (May to September), with mean rainfall of 22 to 70 mm. According to the Köppen-Geiger classification, the area has an “Aw” climate type [24]. The landform of the study location is mountainous. In this region, the greatest risk from the presence of *R. microplus* in cattle occurs between early spring (October/November) and late winter (May/June) [6, 23]. Up to five peaks or generations of this tick species can occur each year [6, 15, 23].

Forty-five Simmental cattle with a mean age of 48 months that were naturally infested by *R. microplus* were selected for the study from a herd of 83 animals. Animals with tick counts ≤ 15 and ≥ 35 were not included in the study. The animals did not receive any treatment with antiparasitic drugs in the 70 days before the start of this study. Only animals with a good nutritional status and body score that were free of diseases that could interfere with the tick load on the animals were included in the study. The animals were observed as general physical appearance and behavior, abnormalities in the locomotor system, consumption of food, water, and appearance of urine and feces. Before the beginning of the study, the animals were kept in the same paddock of coast-cross grass (*Cynodon dactylon*) (19.5 ha), with ad libitum forage, water, and mineral supplementation. During the months of May to September (dry season), each animal received 30 kg of corn silage/animal/day due to the scarcity of forage.

Cattle were allocated to experimental groups on Day 0 and randomly assigned to treatments according to a randomized block design. Block formation was performed based on the arithmetic mean of the number of female ticks (measuring 4.5–8.0 mm in length) counted on three consecutive days (days − 3, − 2, and − 1), as recommended by Wharton and Utech (1970), without multiplying by two [25]. The animals were distributed into 15 blocks containing three cattle each, which were randomly assigned to one of the treatment groups (T01, T02, and T03) within each block. Each group was separated into a different paddock (with each paddock measuring approximately 6.5 hectares), where they remained throughout the experiment. The stocking rates at the beginning of the study for T01, T02, and T03 in the experimental areas were 2.46, 2.72, and 2.56 animal units per hectare (animals/ha), respectively.

### Strategic treatment and rescue protocols adopted to control *R. microplus*

All groups contained 15 animals, and the products were administered according to the manufacturer’s recommendation. The cattle in Groups T01 and T02 were subjected to different protocols of strategic treatments with a pour-on formulation containing 2.5 mg/kg fluralaner (5% Exzolt^®^—MSD Animal Health). The control group (T03) received palliative treatment with a spray formulation (125 ppm alpha-cypermethrin + 400 ppm ethion + 212 ppm chlorpyrifos—Potenty^®^, MSD Saúde Animal) when necessary. To select the commercial product for T03, the population of *R. microplus* involved in the experiment underwent to an adult immersion test (AIT), as recommended by Drummond et al. [26], using different commercial spraying products indicated for lactating cows. Based on the results of the AIT, the commercial association, Potenty^®^, MSD Saúde Animal, was chosen.

For the animals in T01, strategic treatments were performed every 42 days [27], regardless of the degree of infestation, starting in mid-spring (November 2020) until late autumn (June 2021), with treatments performed on Days 0, + 42, + 84, + 126, + 168, and + 210, using the pour-on formulation containing fluralaner.

The cattle in T02 received the first treatment with fluralaner on Day 0 of the study (mid-spring); however, for retreatment, the methodology described by Nicaretta et al. [15], which involved a weekly visual evaluation of the size of ticks (female *R. microplus* < 4 mm) attached between the hind legs and dewlaps of cattle (preferential site of tick attachment), was adopted. Cattle in the T02 group were retreated with the formulation containing fluralaner only when ticks < 4 mm were observed on ≥ 30% (5/15) of the animals in this group [15]. If the mean count of *R. microplus* on cattle in Groups T01 and T02 was ≥ 30 as of June 2021, all animals in the group received palliative treatment with a spray formulation. Before each treatment with fluralaner in both groups (T01 and T02), on the treatment dates, the animals were weighed to calculate the volume of product to be administered.

For cattle kept as controls (T03), all 15 animals in the group received palliative treatment with the spray formulation when the mean tick count of the group was ≥ 30, according to the criterion established by Gomes et al. [13, 18]. It is important to note that this palliative treatment of animals in the T03 group was performed for animal welfare reasons and did not affect the life cycle of the tick because female *R. microplus* ≥ 4 mm in length undergo rapid final engorgement and most detach from cattle within 24 h [25]. Thus, it was possible to observe the infestation of cattle in the T01 and T02 groups by the cattle tick throughout the experimental period.

### Counts of *R. microplus* on animals and in the pasture

Counts of female *R. microplus* between 4.5 and 8.0 mm in length on the left side of each animal, without multiplying by two, were performed weekly by the same person and at the same time according to the methodology proposed by Wharton et al. [25]. Counts were performed on days + 7, + 14, + 21, + 28, and every 7 days until day + 343.

In the pasture, larval counts were performed by the flannel drag technique. A cotton flannel (75 × 100 cm) was dragged over the pasture, according to the methodology described by Nicaretta et al. [28]. Larval counts were performed on Days 0, 30, and 60 and every 30 days until Day 330, always in the first fortnight of each month. In each paddock, where the groups of animals were placed, four flannels were used during dragging. Each flannel was dragged for 300 linear meters; that is, in each picket, dragging was performed for 1200 linear meters. In the event of rain on the larval count days, an interval of 7 days was adopted prior to the next test to avoid the decrease in the number of larvae after the rains [29]. All larvae recovered from each flannel were stored in a freezer (− 20 ℃) for 10 min to reduce their motility; with the aid of a surgical aspirator, quantification of the larvae was performed. Next, the larvae were placed in flasks of 70% ethanol for later identification at the genus level according to Clifford & Anastos [29], and the results were later extrapolated to the tick species present in the pastures.

### Measurement of rainfall, temperature, and relative humidity

Records of rainfall, temperature, and relative humidity were obtained on the farm. A rain gauge (Incoterm 4755) was used to collect precipitation data, and a HOBO data logger (U2300) was used to record ambient temperature and relative humidity. Daily measurements of the maximum, mean, and minimum environmental temperature (°C), relative humidity (%), and total precipitation (mm) were obtained throughout the study period.

### Data analysis

All analyses were performed using Statistical software, version 12 [30]. The counts of female *R. microplus* on animals and of larvae in pastures did not meet the requirements of normality and homogeneity of variance, even after log-transforming the data using log (count + 1); therefore, the nonparametric Kruskal-Wallis test was used.

The monthly mean values of the climatic variables, namely, maximum, minimum, and mean temperature and relative humidity, in addition to total rainfall, were descriptively assessed figures.

## Results

No signs of abnormalities or adverse events were observed in the animals before and after administration of the acaricides (fluralaner and alpha-cypermethrin + ethion + chlorpyrifos). Furthermore, no cattle died, and no concomitant medication was administered during the study.

**Fig. 1:**
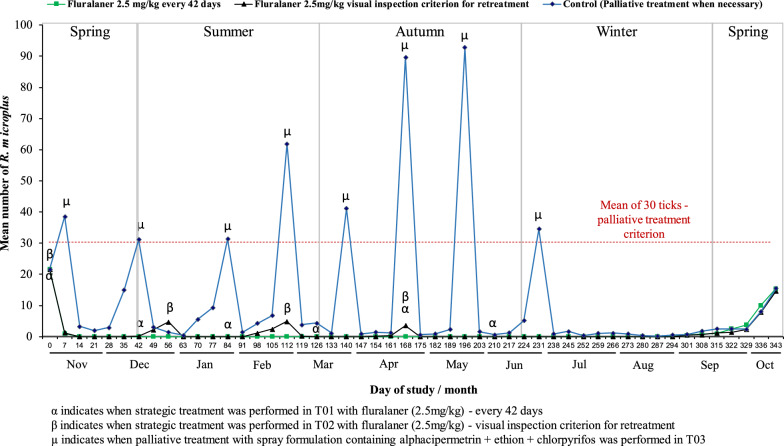
Mean counts of *Rhipicephalus microplus* females (4.5–8 mm in length) parasitizing cattle for 343 days of different strategic control protocols. Legend: α = Day that cattle from T01 received fluralaner (2.5 mg/kg) pour-on—every 42 days between November 2020 to June 2021; β = Day that cattle from T02 received fluralaner (2.5 mg/kg) pour-on—after the treatment on Day 0, the retreatments were performed when ticks < 4 mm were visualized in ≥ 30% (5/15) of the animals from this group; µ = day that cattle from T03 received a palliative treatment with spray, when the mean tick count of this group was ≥ 30

The number of acaricide treatments varied in each group over 12 months. Six applications were performed in Group T01: Day 0 (November 2020, mid-spring), Day +42 (December 2020, late spring), Day + 84 (January 2021, early summer), Day +126 (March 2021, late summer), Day +168 (April 2021, early autumn), and Day +210 (June 2021, late autumn). In Group T02, four treatments were performed: Day 0 (November 2020, mid-spring), followed by three reapplications on Days +56 (December 2020, early summer), +112 (February 2021, late summer) and +168 (April 2021, late autumn). It was not necessary to perform any palliative treatment in Groups T01 and T02 throughout the experimental period (Fig. 1).

In the control group (T03), it was necessary to perform eight palliative acaricide treatments over 12 months with the spray formulation. The treatments performed on cattle belonging to this group occurred on Days +7 (November 2020, mid-spring), +42 (December 2020, late spring), +84 (January 2021, early summer), +112 (February 2021, mid-summer), +140 (March 2021, early autumn), + 168 (April 2021, mid-autumn), + 196 (May 2021, late autumn), and +231 (June 2021, beginning of winter) (Fig. 1).

When comparing the mean counts of female *R. microplus* on the cattle, after Day 0, the animals that were subjected to different control strategies with fluralaner (T01 and T02) showed lower mean tick counts (*p* ≤ 0.05) than the control group (T03) on 28 and 27 of the 49 evaluated dates, respectively. From Day +259 (July 2021, midwinter) to Day +343 (October 2021, early spring), there was no difference (*p* > 0.05) in the mean tick counts among the three groups. It should be noted that in Groups T01 and T02, after the first treatment with fluralaner, all counts were close to 0 throughout the study period (Table 1).

**Table 1:** Mean counts of *Rhipicephalus microplus* females (between 4.5 and 8 mm) in cattle submitted to different schemes and strategic control with fluralaner pour-on and control with an association of alphacypermethrin + ethion + chlorpyrifos spray

Day of study	Date that chemical treatment was performed in the respective group	Experimental groups						Value Test^2^	Pr > KW^3^
		T01: fluralaner (2.5 mg/kg) every 42 days		T02: Fluralaner (2.5 mg/kg) visual inspection for retreatment		T03: control (treatment when necessary)			
		Mean ^1^ count ± standard deviation/median/range		Mean ^1^ count ± standard deviation/median/range		Mean ^1^ count ± standard deviation/median/range			
0*	T01 and T02	21.6 ± 3.4	21.0 (17–30) A	21.6 ± 3.3	20.7 (18–30) A	21.6 ± 3.1	21.7 (17–29.7) A	0.15	0.9287
7	T03	1.0 ± 1.1	1.0 (0–3) B	1.3 ± 1.5	0.0 (0–4) B	38.6 ± 9.0	36.0 (26–32) A	30.42	< 0.0001
14		0.0 ± 0.0	0.0 (0–0) B	0.0 ± 0.0	0.0 (0–0) B	3.3 ± 3.4	2.0 (0–10) A	27.78	< 0.0001
21		0.0 ± 0.0	0.0 (0–0) B	0.0 ± 0.0	0.0 (0–0) B	1.9 ± 1.9	3.0 (0–5) A	18.83	0.0001
28		0.0 ± 0.0	0.0 (0–0) B	0.0 ± 0.0	0.0 (0–0) B	2.9 ± 2.7	4.0 (0–7) A	21.68	< 0.0001
35		0.0 ± 0.0	0.0 (0–0) B	0.0 ± 0.0	0.0 (0–0) B	15.0 ± 7.5	14.0 (6–34) A	41.71	< 0.0001
42	T01 and T03	0.0 ± 0.0	0.0 (0–0) B	0.1 ± 0.5	0.0 (0–2) B	31.1 ± 10.6	31.0 (14–56) A	40.13	< 0.0001
49		0.0 ± 0.0	0.0 (0–0) B	2.3 ± 3.5	0.0 (0–11) A	3.0 ± 3.0	3.0 (0–8) A	13.59	0.0011
56	T02	0.0 ± 0.0	0.0 (0–0) B	4.7 ± 4.1	4.0 (0–14) A	1.5 ± 1.8	1.0 (0–6) AB	19.23	0.0001
63		0.0 ± 0.0	0.0 (0–0) A	0.0 ± 0.0	0.0 (0–0) A	0.5 ± 1.2	0.0 (0–4) A	4.09	0.1293
70		0.0 ± 0.0	0.0 (0–0) B	0.0 ± 0.0	0.0 (0–0) B	5.6 ± 4.3	6.0 (0–13) A	31.03	< 0.0001
77		0.0 ± 0.0	0.0 (0–0) B	0.0 ± 0.0	0.0 (0–0) B	9.3 ± 4.8	9.0 (3–18) A	41.78	< 0.0001
84	T01 and T03	0.0 ± 0.0	0.0 (0–0) B	0.0 ± 0.0	0.0 (0–0) B	31.3 ± 15.5	33.0 (10–64) A	41.70	< 0.0001
91		0.0 ± 0.0	0.0 (0–0) B	0.0 ± 0.0	0.0 (0–0) B	1.5 ± 1.8	1.0 (0–6) A	21.67	< 0.0001
98		0.0 ± 0.0	0.0 (0–0) B	1.2 ± 2.6	0.0 (0–8) B	4.3 ± 3.4	4.0 (0–10) A	20.94	< 0.0001
105		0.0 ± 0.0	0.0 (0—0) B	2.4 ± 4.0	0.0 (0–13) B	6.8 ± 4.4	6.0 (0–13) A	22.33	< 0.0001
112	T02 and T03	0.0 ± 0.0	0.0 (0–0) B	4.9 ± 6.0	2.0 (0–18) B	61.9 ± 37.0	46.0 (9–125) A	35.66	< 0.0001
119		0.0 ± 0.0	0.0 (0–0) B	0.2 ± 0.6	0.0 (0–2) B	3.8 ± 3.3	4.0 (0–9) A	20.40	< 0.0001
126	T01	0.0 ± 0.0	0.0 (0–0) B	0.0 ± 0.0	0.0 (0–0) B	4.3 ± 3.1	4.0 (0–10) A	34.44	< 0.0001
133		0.0 ± 0.0	0.0 (0–0) B	0.0 ± 0.0	0.0 (0–0) B	1.1 ± 1.2	1.0 (0–4) A	21.71	< 0.0001
140	T03	0.0 ± 0.0	0.0 (0–0) B	0.0 ± 0.0	0.0 (0–0) B	41.1 ± 36.7	35.0 (4–156) A	41.70	< 0.0001
147		0.0 ± 0.0	0.0 (0–0) A	0.0 ± 0.0	0.0 (0–0) A	0.9 ± 1.9	0.0 (0–6) A	6.28	0.0734
154		0.0 ± 0.0	0.0 (0–0) A	0.1 ± 0.3	0.0 (0–1) A	1.5 ± 2.1	0.0 (0–6) A	13.23	0.0613
161		0.0 ± 0.0	0.0 (0–0) B	0.3 ± 0.7	0.0 (0–1) B	1.3 ± 1.0	1.0 (0–3) A	19.09	0.0001
168	T01, T02 and T03	0.0 ± 0.0	0.0 (0–0) B	3.6 ± 7.4	0.0 (0–21) B	89.7 ± 61.7	68.0 (19–241) A	36.18	< 0.0001
175		0.0 ± 0.0	0.0 (0–0) A	0.0 ± 0.0	0.0 (0–0) A	0.7 ± 1.0	0.0 (0–3) A	13.46	0.0012
182		0.0 ± 0.0	0.0 (0–0) B	0.0 ± 0.0	0.0 (0–0) B	0.9 ± 0.8	1.0 (0–2) A	21.73	< 0.0001
189		0.0 ± 0.0	0.0 (0–0) B	0.0 ± 0.0	0.0 (0–0) B	2.3 ± 2.5	2.0 (0–10) A	10.31	< 0.0001
196	T03	0.0 ± 0.0	0.0 (0–0) B	0.0 ± 0.0	0.0 (0–0) B	92.8 ± 65.0	85.0 (24–265) A	41.69	< 0.0001
203		0.0 ± 0.0	0.0 (0–0) B	0.0 ± 0.0	0.0 (0–0) B	1.6 ± 2.1	1.0 (0–8) A	21.71	< 0.0001
210	T01	0.0 ± 0.0	0.0 (0–0) A	0.0 ± 0.0	0.0 (0–0) A	0.7 ± 1.0	0.0 (0–3) A	13.46	0.0654
217		0.0 ± 0.0	0.0 (0–0) A	0.0 ± 0.0	0.0 (0–0) A	1.3 ± 3.1	0.0 (0–12) A	13.46	0.0743
224		0.0 ± 0.0	0.0 (0–0) B	0.0 ± 0.0	0.0 (0–0) B	5.1 ± 3.7	5.0 (0–12) A	04.38	< 0.0001
231	T03	0.0 ± 0.0	0.0 (0–0) B	0.0 ± 0.0	0.0 (0–0) B	34.6 ± 24.4	29.0 (5–74) A	41.71	< 0.0001
238		0.0 ± 0.0	0.0 (0–0) A	0.0 ± 0.0	0.0 (0–0) A	0.9 ± 1.1	0.0 (0–3) A	16.09	0.0571
245		0.0 ± 0.0	0.0 (0–0) B	0.0 ± 0.0	0.0 (0–0) B	1.7 ± 1.5	2.0 (0–5) A	27.79	< 0.0001
252		0.0 ± 0.0	0.0 (0–0) A	0.0 ± 0.0	0.0 (0–0) A	0.4 ± 0.8	0.0 (0–3) A	8.57	0.0838
259		0.0 ± 0.0	0.0 (0–0) A	0.0 ± 0.0	0.0 (0–0) A	1.1 ± 3.1	0.0 (0–12) A	10.98	0.1041
266		0.0 ± 0.0	0.0 (0—0) A	0.0 ± 0.0	0.0 (0–0) A	1.2 ± 3.6	0.0 (0–14) A	8.56	0.1138
273		0.0 ± 0.0	0.0 (0—0) A	0.0 ± 0.0	0.0 (0–0) A	0.9 ± 3.1	0.0 (0–12) A	6.28	0.1433
280		0.0 ± 0.0	0.0 (0–0) A	0.0 ± 0.0	0.0 (0–0) A	0.4 ± 0.7	0.0 (0–2) A	8.57	0.0738
287		0.0 ± 0.0	0.0 (0–0) A	0.0 ± 0.0	0.0 (0–0) A	0.2 ± 0.4	0.0 (0–1) A	6.29	0.0632
294		0.1 ± 0.3	0.0 (0–1) A	0.0 ± 0.0	0.0 (0–0) A	0.5 ± 0.7	0.0 (0–2) A	10.42	0.1555
301		0.5 ± 0.7	0.0 (0–2) A	0.5 ± 1.1	0.0 (0–4) A	0.7 ± 0.8	1.0 (0–2) A	1.65	0.4386
308		0.9 ± 0.8	1.0 (0–2) A	0.7 ± 0.9	0.0 (0–2) A	1.8 ± 1.5	2.0 (0–5) A	6.24	0.0144
315		1.0 ± 0.8	1.0 (0–2) A	1.2 ± 0.9	1.0 (0–3) A	2.5 ± 1.9	2.0 (0–5) A	5.54	0.0626
322		2.5 ± 2.1	2.0 (0–4) A	1.4 ± 1.5	1.0 (0–5) A	2.5 ± 3.0	2.0 (0–12) A	2.39	0.3031
329		3.7 ± 1.7	4.0 (0–6) A	2.3 ± 2.9	2.0 (0–12) A	2.4 ± 1.9	2.0 (0–6) AB	7.64	0.0719
336		10.1 ± 8.0	9.0 (1–24) A	7.8 ± 5.9	5.0 (0–20) A	8.1 ± 6.9	9.0 (0–19) A	0.69	0.7084
343		15.4 ± 8.0	17.0 (0–26) A	14.6 ± 5.7	15.0 (4–24) A	15.5 ± 8.0	14.0 (5–32) A	0.38	0.8261

^*^Means of tick counts between days − 3, − 2 and − 1

1: Means followed by the same capital letter in the line do not differ from each other (*P* ≤ 0.05)

2: value of the Kruskal-Wallis test

3: Probability of significance of the Kruskal-Wallis Test

**Fig. 2:**
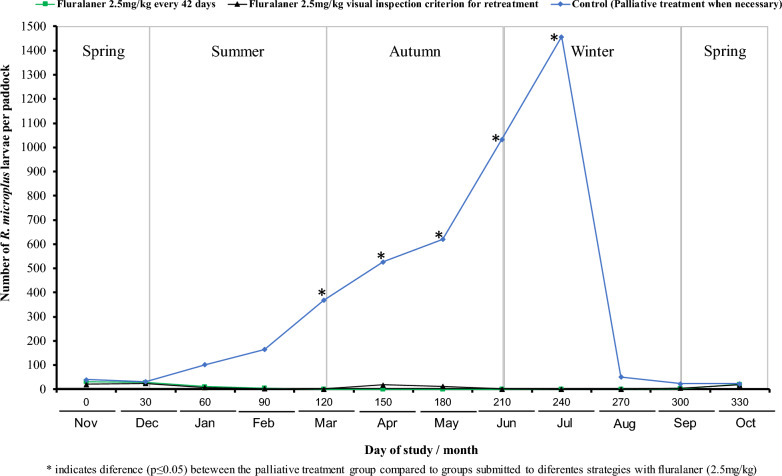
Total counts of *Rhipicephalus microplus* larvae in paddocks occupied by animals from different strategic control protocols with fluralaner (2.5 mg/kg) pour-on and a palliative treatment with spray formulation. *On the same date, tick larva count differs significantly (*p* ≤ 0.05)

All the larvae collected were identified as genus *Rhipicephalus*. The total number of *R. microplus* larvae collected in the paddock where the T01 cattle remained between November 2020 (mid-spring) and February 2021 (mid-summer) ranged from 5 to 32. Larvae were found in pastures between March (late summer) and August 2021 (midwinter). In the paddock where the animals in the T02 group were kept, in November and December 2020 (mid and late spring), 21 and 25 larvae were collected, respectively. From January to September 2020 (from summer until the end of winter), ≤ 19 larvae were observed per month in this paddock (Fig. 2).

In the paddock where the animals were kept as controls, the *R. microplus* larvae counts increased between mid-spring (November 2020; *n* = 41) and the beginning of winter (July 2021; *n* = 1458; dragging was performed on 5 July). In the winter of 2020 (August), after two frosts that occurred because of low temperatures on July 8 (1.9 ℃) and July 21 (2.7 ℃), the number of larvae found in this paddock decreased considerably (52 larvae). Larval counts in the paddock pastures used to house the animals subjected to different treatments with fluralaner (T01 and T02) did not differ (*p* > 0.05) during the 12 months of the study. On the other hand, the total number of larvae in the paddocks of these two groups was lower (*p* ≤ 0.05) than the number of larvae in the paddock used to house the control animals (T03) from March to July 2021 (late summer to mid-winter) (Fig. 2).

**Fig. 3:**
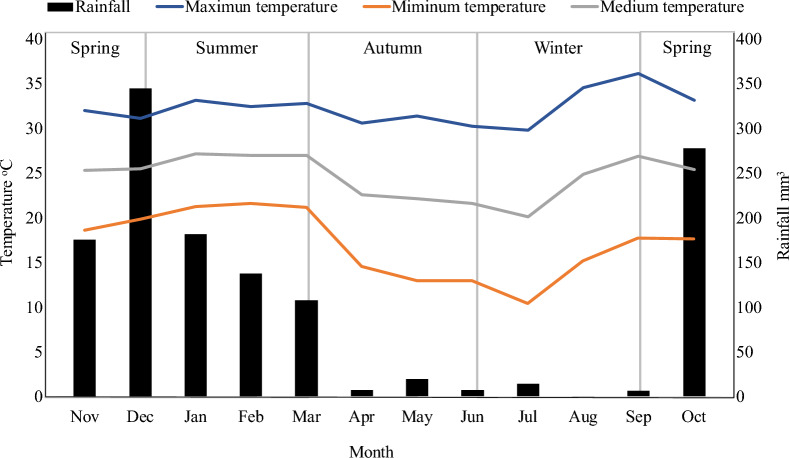
Monthly mean values of temperature (ºC) maximum, average, minimum, and historical rainfall (mm) from November 2020 to October 2021

**Fig. 4:**
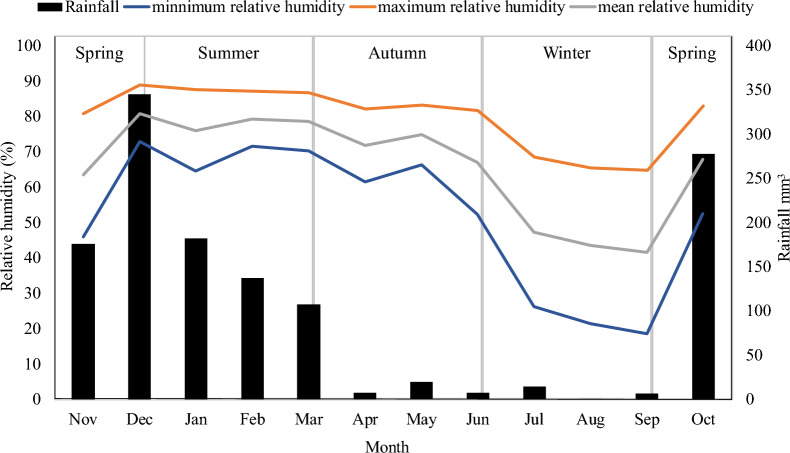
Monthly mean values of relative humidity (%) maximum, average, minimum, and historical rainfall (mm) from November 2020 to October 2021

Figures 3 and 4 show the mean monthly rainfall, environment temperature (maximum, minimum and mean), and relative humidity (maximum, minimum, and mean) during November 2020 to October 2021.

## Discussion

This article presents unprecedented results on different cattle tick control strategies using pour-on fluralaner (2.5 mg/kg). The mean number of ticks on the taurine cattle kept in a tropical region and treated with fluralaner during 1 year of study remained zero on most of the observation days. In contrast, the animals in the control group received palliative treatment with the spray formulation (2 organophosphates + 1 pyrethroid) according to tick load. Regarding pasture infestation, the number of larvae was lower in the paddocks that housed the animals that were treated with fluralaner than in the paddock that housed the animals kept as the control. It was possible to decrease the number of chemical treatments applied over a year and increase the intervals between applications by performing visual inspections once a week. In the group of cattle that received fluralaner every 42 days (T01), six acaricide treatments were performed. For the cattle in the T02 group, based on the visual inspections, four treatments were performed over a year, with an interval of 56 days between applications.

Although it is not known exactly how many treatments are required to prolong the shelf life of a formulation in a given property, it is clear that the resistance of *R. microplus* to a particular chemical is directly linked to the frequency and mode (under- or overdose) of use of this formulation against this ectoparasite [2, 14, 31–35]. In Mexico, Rodriguez-Vivas et al. [36] reported that there is a greater likelihood of populations of *R. microplus* being resistant to organophosphates and pyrethroids when more than six acaricide applications are performed per year. In cattle farms in Veracruz, Mexico, Fernandez-Salas et al. [37] reported that farmers who used macrocyclic lactones more than four times a year were more likely to have ivermectin-resistant populations of *R. microplus*. In Australia, Sutherst [38] and Jonsson et al. [39] found a higher likelihood of having populations of *Rhipicephalus australis* that were resistant to cypermethrin, deltamethrin and flumethrin when more than six acaricide treatments with formulations containing these active ingredients were performed per year compared to the likelihood when four or five treatments were performed per year. From a practical point of view, retreating animals based on the visual inspection approach described in this study may be an interesting strategy in the field. In addition to reducing the mean cost of treatment with acaricides for 12 months on the farm, this approach may also lead to lower exposure of the *R. microplus* population to fluralaner during this period, which could help maintain the efficacy of this chemical over time. In any case, future studies should be performed to validate the hypothesis that the duration of fluralaner efficacy depends on the number of treatments performed during a given period.

It is important to note that this study does not aim to recommend that only fluralaner be used in strategic control. The exclusive use of fluralaner in the present study aimed to understand how this new molecule performs in the control of *R. microplus* over 1 year in a region with high levels of challenge by this tick species (with the occurrence of up to five generations per year) and animals of European origin. There is evidence from two previous studies that alternating chemicals with each treatment is better than using the same chemical base sequentially due to selection pressure [40, 41]. One was a field study conducted by Jonsson et al. [42] alternating amitraz and spinosad to treat *R. australis*. An in vitro experiment was conducted by Thulner et al. [40], with deltamethrin and coumaphos, in a field population of *R. microplus* from Costa Rica. If the alternation of chemicals is the strategy to be adopted by technicians and veterinarians in the field, the new fluralaner molecule may be another alternative to add to the rotation of active ingredients used to achieve the strategic control of *R. microplus*.

Another important aspect that should be highlighted is the possibility of postponing or anticipating the reapplication of acaricides of a certain formulation within a strategic control protocol [4, 7, 18, 28]. Because fluralaner is a new molecule, the levels of residual efficacy remain high (≥ 95%). After this new molecule has been used for a certain time, the susceptibility of *R. microplus* to the molecule will tend to decrease, which may mean that this strategy can no longer be used because of the selection pressure on the population of ticks at the site. An example is the scenario observed for fluazuron: when this molecule arrived on the market, it had a residual efficacy ≥ 90% for approximately 60 to 70 days. After years of use, the residual efficacy of this active in the control of *R. microplus* decreased to 28 to 42 days [34, 35]. When the period of residual efficacy of a formulation decreases in the field, the need for acaricide treatments should be anticipated, and it is necessary to change the previously established/recommended protocol [28].

Both strategies adopted with 2.5 mg/kg of pour-on fluralaner in the present study were sufficient to interrupt the parasitic phase of *R. microplus* and provide a minimum larval infestation (≤ 25 larvae/patch) in the pickets throughout the year; in the countryside, this is popularly known as “cleaning the pastures.” This effect occurred in the T01 group because the animals were retreated every 42 days; a negligible infestation of *R. microplus* was observed on the day of retreatment (mean counts of zero females ≥ 4.5–8 mm), as described in another study [26]. In T02, this effect was observed after performing visual inspections as a treatment criterion. It is possible that acaricide retreatment based on tick length prevents the detachment of fully engorged females from the host to the pasture and consequent oviposition and larval hatching. It should be noted that partially engorged females (≥ 5–6 mm in length) undergo rapid final engorgement (8–11 mm long) at night and detach from the host in the morning [25, 42]. Thus, treatment prior to this stage breaks the cycle, considerably reducing the chance of females reaching the soil and starting the oviposition process. Similar results were obtained by Nicaretta et al. [28] using the same methodology. It is important to highlight that use the visual inspection as a criterion of acaricide treatment provides positive results, but when more than one person is involved in these evaluations, the results can be subjective. Therefore, if the decision is made to employ this strategy, it is important to consider this aspect to ensure that the visual inspection-based strategy aligns with positive results.

The counts of *R. microplus* on the animals and in the pastures for the control group (T03) demonstrate that the ectoparasite in question challenged the 45 cattle in the study and help to reinforce that the results found for the groups treated with fluralaner are inherent to the efficacy of the pour-on formulation containing this new molecule. The mean tick counts of the control animals that were treated palliatively with the spray formulation when necessary (T03) and pastures that housed the control animals were higher than those observed for the animals treated with fluralaner (T01 and T02). The abrupt decrease in the larval counts in pastures in the winter of 2021, between July (1450 larvae count performed on July 4) and August (52 larvae), in the paddock of control animals (T03), is possibly related to the low minimum temperature values recorded in July 2021 (1.9° and 2.7 ℃) between July 8 and 21, respectively. On those same days, frost occurred, with the presence of ice in the pastures, leading to the death of plants or their parts (leaves, branches, fruits) since low temperatures can cause freezing of plant tissues, with or without the formation of ice on the plants [43]. In addition, it is known that relatively high temperatures and humidity are required for the survival of *R. microplus* during the nonparasitic phase [14].

In the region where the present study was conducted, the highest population peaks of *R. microplus* occurred in the period from October/November (spring) to June/July (late autumn/early winter), termed the “tick season” [6]. When the life cycle of this ectoparasite is not interrupted by appropriate chemical strategies, the tendency is that in the first generations of the tick between September/October, which corresponds to early spring, the degree of infestation of animals and pastures by *R. microplus* is lower than that at the end of the tick season in June/July (late autumn/early winter). This is due to a cumulative effect initiated by the females that develop in the first generation (“spring raise”, the beginning of spring); with each new generation, a greater number of females arrive in the soil and consequently a greater number of eggs and larvae. In practice, this may cause the interval between treatments to decrease at the end of the “tick season,” with treatments coinciding with the last generations in autumn and winter because of the higher degree of infestation of animals during this period [4, 7, 18]. In the present study, this phenomenon occurred in the animals of the control group (T03), with the first three treatments performed in November 2020, December 2020, and January 2021 (mid-spring to early summer), occurring with an interval of 35 to 42 days between applications; treatments at the end of the tick season in March, April, and May 2021 were performed with an interval of 28 days between applications. Notably, the decrease in the interval between applications was not observed in either of the two groups that received pour-on fluralaner by different strategies. This finding highlights this molecule, which belongs to the isoxazoline class, as an excellent and promising tool in the strategic control of cattle ticks under field conditions.

After the end of each year’s tick season, which can vary between May and August depending on the region of Brazil, a “reset” in the tick population occurs, and this is determined by abiotic factors, especially temperature and humidity. In some regions of South America such as Argentina and southern Brazil, field and laboratory studies have shown that minimum temperatures may cause a decrease in this ectoparasite in the environment and consequently on animals [14, 17, 44–46], as observed in the present study. On the other hand, the minimum temperature values in parts of the southeastern and center-west regions of Brazil are milder than those in the south region. Thus, the high saturation deficit, which occurs during the dry period of the year in months with high temperatures (± 30 ℃) and low relative humidity (≤ 35%), is the abiotic factor responsible for the decrease in the population *R. microplus* in the environment and consequently on animals [15, 47]. In the present study, the animals kept as controls (T03) received the last acaricide application in the last week of June, 7 days before the first frost. This fact may explain why tick infestation on animals and in the environment decreased between the months of July/August and October 2021 in the control group (T03) and remained low in the groups that received the different control strategies with fluralaner (T01 and T02).

It is possible that fewer annual treatments with fluralaner are required in regions where the cattle tick completes fewer generations per year, and this molecule may be another option for rotational control in these regions. For example, in Argentina, where the dynamics of *R. microplus* are characterized by three annual generations [48], a “generational” strategic protocol with three treatments, each with a different drug (ivermectin, fluazuron or fipronil), was sufficient to control *R. microplus* [49–52]. However, for bulls raised in southeastern and midwestern Brazil, the scenario is different. Populations of *R. microplus* are resistant to different chemical classes [14, 34, 35, 52–56]. In addition, currently, there are regions with five population peaks or five generations of ticks per year, a fact that implies the need to perform more chemical treatments during the year [14, 15, 34]; thus, generational control of cattle ticks on taurine cattle is impracticable in these regions of Brazil [15].

Notably, the number of treatments may be influenced by the resistance profiles of *R. microplus* populations in different locations. Therefore, before adopting any strategy with fluralaner or any other formulation, one should consider the biology and epidemiology of *R. microplus* in the region to ensure the rational use of antiparasitic drugs. Calculation of the number of generations of this ectoparasite that occur each year is essential to establishing the best strategy.

## Conclusions

The different strategic treatment protocols performed with pour-on fluralaner (2.5 mg/kg) over a year in taurine cattle in a tropical region with a history of up to five annual generations of cattle ticks were effective, maintaining levels of *R. microplus* infestations in animals and in pastures close to zero in most of the study. On the other hand, in the control group, cattle that received palliative treatment with a spray formulation based on the tick counts, the mean tick counts on the animals and the total larvae in the pasture were 92.8 and 1458, respectively. The cattle treated with fluralaner every 42 days received six acaricide treatments in 1 year, while the animals treated with fluralaner and retreated following visual inspection received four acaricide treatments. Depending on the retreatment criterion adopted, the number of applications per year may be lower, resulting in a reduction in the mean cost of acaricide treatment per year and lower exposure of *R. microplus* populations to the active ingredient, resulting in lower resistance and selection pressure.

## Data Availability

The data used to obtain the findings of this study are available from the corresponding author upon request.

## Abbreviations

T01: Fluralaner (2.5 mg/kg) every 42 days
T02: Fluralaner (2.5 mg/kg) visual inspection for retreatment
T03: Control (Treatment when necessary)
ECUA: Ethics Committee on the Use of Animals
UFG: Federal University of Goias
